# What is the value of disease expenditure studies? An argument for an international database of spending estimates

**DOI:** 10.1186/s44263-023-00021-6

**Published:** 2023-10-23

**Authors:** Emily Bourke, Samantha Grimshaw, Tony Blakely

**Affiliations:** Population Interventions, Centre for Epidemiology and Biostatistics, Melbourne School of Population and Global Health, University of Melbourne, Melbourne, Australia

## Abstract

Based on a recent study on disease-specific health spending by age, sex, and type of care in Norway, we argue for the need to improve disease spending estimates and to create a database similar to the Global Burden of Disease Study.

## Background

Undertaking a disease expenditure study that captures all health system spending and accounts for comorbidities is, put mildly, no simple task. The recent analysis by Kinge et al. [1] in *BMC Medicine* uses detailed Norwegian data to decompose the National Health Accounts into the conditions that are being treated and managed through the health and long-term care systems. This is a robust analysis that was able to leverage off the rich diagnostic information in the datasets used and generally adjusts for comorbidity costs and, in addition to disease expenditure, was able to include ‘well care’ (spending on preventive and routine care and check-ups, such as managing a healthy pregnancy) and costs of treatment of risk factors that are managed medically (such as hypertension and hyperlipidaemia). The limitations of the study are common with other disease expenditure studies—mainly, some missing or inadequate diagnosis codes, and informal care (such as care provided by family members) not being captured. The findings are interesting—almost 1 in 5 Norwegian Krone that are spent in the health system are spent managing mental and substance abuse disorders. However, these same conditions account for only 8% of the disease burden in disability-adjusted life years in Norway [2].

## Comparing across countries

Understanding how national health expenditure is disaggregated by disease is a valuable contribution to evidence-based policymaking and planning, particularly given population ageing and concerns of health system sustainability in the future. Comprehensive disease expenditure analyses have been undertaken in several other high-income countries—namely, the United States (US) [3], Switzerland [4], and Australia [5], and also New Zealand with different methods [6]. These analyses are generally comparable as they all based their disease classifications off the Global Burden of Disease (GBD) condition list (or the Australian equivalent, for that country). Comparisons of disease spending and analysis of areas of difference can enable countries to learn from each other and improve health system efficiency. For example, long-term care includes a much larger health component in Norway than in these other countries, and mental health care spending is substantially higher in Norway (20.7%) than in the US (6.7%), Australia (7.7%), and Switzerland (10.6%). Future research could usefully address whether this contributes to higher quality of life in older age, or better outcomes for those with mental health conditions, which could then contribute to policy changes in other countries.

Data on the economic and health system impacts of diseases across countries is crucial to evidence-based health policymaking and planning. However, such analyses are unable to be conducted by most countries due to limited data availability, or lack of analysts (or funding) to do so. Without country-specific information on disease-specific costs to the health system, it is much harder to conduct health economic evaluations of public health policies and interventions—as a major benefit of prevention is reducing disease rates, which in turn reduces disease expenditure in the near-term, freeing up resources to deploy on other health and disease problems [7]. To adequately assess healthcare spending trends and future needs, the world needs to have estimates of what health expenditure would be in the future based on demographic and disease forecasts, assuming the cost basis for disease treatments stays the same—requiring a comprehensive overview of spending by health condition. The next logical step in the field is to create a resource similar to what the GBD study has created for epidemiology metrics [2]—a parallel health expenditure database by country. This would involve a large amount of estimation based on existing data sources (GBD epidemiology and demographic data, national health accounts estimates, and known spending patterns from countries that collect this data).

## Next steps

To generate a global database of country-level health expenditure by disease, it is necessary to ensure consistency among underlying disease expenditure studies in which types of spending are attributed and included (particularly aged care spending pertaining to health), and the perspectives used (government spending or total spending), which are currently inconsistent between countries that have undertaken disease spending analyses. The development of standards for disease costing studies would be an ideal starting point for any broader work to estimate and compare disease spending across countries. Additionally, the dynamic nature of disease incidence and cost differentials by disease phase—incident, prevalent, and last year of life—needs to be factored in for forecasting future health expenditure. The next stage of disease spending estimation and research should expand to try and estimate spending by phase. This will enable dynamic costs to be imputed into a population simulation model to appropriately test these assumptions, and plan accordingly.

While spending information alone is not sufficient to answer the types of questions posed here, the alignment of cost estimates to GBD condition data and Organisation for Economic Cooperation and Development health spending is a solid foundation to start comprehensively evaluating them. This will also enable the modelling of hypothetical changes in disease incidence (or case fatality and remission) rates, allowing to determine priority areas to address for reasons over and above just health gain and health inequality reduction.

This would have enormous value in terms of health system planning and prioritising future budget allocations as populations age. From 2019 to 2050, the global population aged 65 years and older is expected to double in size to 1.5 billion [8], while the total global population is unlikely to increase by more than 30% over the same period [9]. At least one-quarter of the non-communicable disease (NCD) burden is attributable to people aged 70 years and over, while nearly 88% of the disease burden in this age group is due to NCDs [2]. Public health challenges associated with ageing populations include reduced economic growth, increased disabilities, multimorbidity, and increased health spending—all of which will vary by ethnicity, geography, sex, and socioeconomic status.

Yes, populations are ageing. But if they are also getting healthier at a given age, then the morbidity may be—theoretically—shifted out. How this will affect health budgets is an area of large uncertainty—the case could also be made that total health spending is a policy decision and not a pure function of disease burden. Many researchers and policy-makers assume morbidity by age is static going forward, and indeed GBD analyses show that the rate of years lived with disease by age does not change too much over time, although which diseases contribute to that morbidity does change over time [10]. The perpetual question given this context is “Will preventing disease reduce spending in old age?”, or at least free up some resources to deploy elsewhere. This is only possible to answer with integrated disease expenditure data and population simulation models. The dynamic nature of disease development and costs by phase is therefore important to capture in costing projections.

## Conclusions

Strengthening health information systems across countries would improve current challenges related to data availability, quality, and relevance, allowing more countries to generate their own disease expenditure estimates. While these estimates would not provide all the information necessary to reduce disease burden, they are an excellent starting point towards a much bigger goal: a resource that brings together comprehensive health expenditure data and analyses of worldwide trends, similar to the GBD Study. The recently published work on disease spending in Norway by Kinge et al. [1] brings us one step closer to having such consistent estimates.

## Data Availability

Not applicable.
